# Breaking the vicious cycle: The interplay between loneliness, metabolic illness, and mental health

**DOI:** 10.3389/fpsyt.2023.1134865

**Published:** 2023-03-08

**Authors:** Minhal Ahmed, Ivo Cerda, Molly Maloof

**Affiliations:** ^1^Harvard Medical School, Boston, MA, United States; ^2^Adamo Bioscience, Inc., Fernandina Beach, FL, United States

**Keywords:** loneliness, social isolation, metabolic syndrome, mental health, depression, chronic stress, HPA axis, mitochondria

## Abstract

Loneliness, or perceived social isolation, is a leading predictor of all-cause mortality and is increasingly considered a public health epidemic afflicting significant portions of the general population. Chronic loneliness is itself associated with two of the most pressing public health epidemics currently facing the globe: the rise of mental illness and metabolic health disorders. Here, we highlight the epidemiological associations between loneliness and mental and metabolic health disorders and argue that loneliness contributes to the etiology of these conditions by acting as a chronic stressor that leads to neuroendocrine dysregulation and downstream immunometabolic consequences that manifest in disease. Specifically, we describe how loneliness can lead to overactivation of the hypothalamic-pituitary-adrenal axis and ultimately cause mitochondrial dysfunction, which is implicated in mental and metabolic disease. These conditions can, in turn, lead to further social isolation and propel a vicious cycle of chronic illness. Finally, we outline interventions and policy recommendations that can reduce loneliness at both the individual and community levels. Given its role in the etiology of the most prevalent chronic diseases of our time, focusing resources on alleviating loneliness is a vitally important and cost-effective public health strategy.

## Introduction

Even before the coronavirus pandemic limited in-person gatherings and social activities, U.S. Surgeon General Vivek Murthy called the growing “crisis of loneliness” plaguing the country a concerning public health epidemic (1). Loneliness, or perceived social isolation, is the subjective feeling of a mismatch between one’s desired and actual levels of social connection (2). Loneliness differs from social disconnection in that the latter is an objective measure of connectivity, while the former is a subjective state (3, 4). Loneliness is widely recognized as a major risk factor for morbidity and premature mortality (5); by some estimates, lack of social connection increases the odds of death by as much as 50% (5–7). The prevalence of loneliness is alarming: according to a World Health Organization report, in 2021, nearly one-third of U.S. older adults felt lonely frequently (8).

Epidemiological studies have associated loneliness with some of the most pressing public health challenges of our time, namely, the epidemics of chronic diseases like mental illness and the metabolic syndrome (MetS), which includes hypertension, dyslipidemia, obesity, and insulin resistance. Loneliness is associated with an increased risk of developing myriad neuropsychiatric disorders, including major depressive disorder, anxiety disorders, and post-traumatic stress disorder (9–14). For example, by performing cross-lagged analyses on longitudinal data from middle-aged and older U.S. populations, Cacioppo and colleagues demonstrated that loneliness predicted subsequent increases in depressive symptoms, but not vice versa, suggesting that loneliness may play a causal role in the development of depression rather than being a side-effect (14). While most of the research into MetS has focused on the contribution of diet and physical activity, some epidemiological studies have also identified associations between conditions of MetS and loneliness or other measures of social integration (15, 16). Notably, a large longitudinal study revealed that individuals who reported increased levels of loneliness had greater odds of developing MetS, an effect that was partly mediated by depression (17).

Here, we characterize loneliness as a major chronic stressor common to the pathogenesis of both mental and physical disease through neuroendocrine, immune, and ultimately metabolic, dysregulation. Furthermore, we describe how these physiological consequences of loneliness can lead to further isolation in a vicious cycle. To counteract the pathophysiological consequences of loneliness, we suggest strategies and interventions that increase metabolic and mental resilience at an individual level and argue that improving social connection is an effective public health strategy to alleviate loneliness and the chronic diseases it may contribute to.

## Loneliness leads to pathophysiological changes: A chronic stress model

From an evolutionary perspective, social connections are important for various survival-related behaviors, including foraging, protection against predation, and reproduction. Evolutionary pressures on social species have shaped our brains and endocrine systems, in part to promote cooperation and be reactive against social isolation. Accordingly, loneliness is thought to have evolved as an alarm signal, akin to hunger or thirst, to seek out social contact and promote survival (2, 18, 19). While adaptive in acute settings, chronic activation of this stress response by prolonged feelings of social isolation—or, chronic loneliness, hereafter referred to as loneliness—has long-term costs. As such, loneliness acts as a chronic stressor that taxes the neuroendocrine, immune, and metabolic systems of the body and leads to the chronic physical and mental diseases associated with social isolation.

Although the chronic stress of loneliness might lead to poor metabolic and mental health through numerous pathways—including dysregulation of sympathoadrenal or autonomic nervous system function [for reference, see Vitale and Smith (20)]—evidence for the effect of loneliness on these systems remains relatively scant and inconsistent (21). Here, we focus on the overactivation of the hypothalamic-pituitary-adrenal (HPA) axis and its downstream immunometabolic consequences, particularly as they relate to mitochondrial function dysregulation.

Lastly, MetS is a multi-parameter condition capturing the high-frequency co-occurrence of metabolic risk factors for type 2 diabetes mellitus and cardiovascular disease. Though the disorders of MetS have vastly different clinical presentations in isolation, all likely arise from common pathophysiological pathways including neurohumoral activation, insulin resistance, and chronic inflammation. For simplicity, and given their common pathophysiology and high frequency of comorbidity, here we do not explore differential pathways or interventions for each disorder of MetS, except when consideration of a condition in isolation serves an illustrative purpose.

### Risk factors for loneliness

Various genetic, environmental, and demographic factors can increase an individual’s susceptibility to loneliness. Evidence from twin studies suggest that loneliness is a moderately heritable trait, with estimates of heritability ranging from 0.4–0.5 (22). A recent analysis of large-scale genome-wide association study data not only identified unique genetic loci associated with loneliness, but also found shared genetic overlap between risk factors for loneliness, cardiovascular disease, and severe mental disorders (22). Functional analyses of these loci implicated biological processes related to the brain, immune system, and metabolism, and suggested that genetic risk for loneliness may increase the risk for both cardiovascular disease and mental illness (23).

Beyond genetics, marriage, having children, higher levels of education, and a larger number of siblings may protect against loneliness, while having a male gender, chronic work or social stress, and physical health symptoms may put one at greater risk (21, 24, 25). Though prevalent across all age groups, loneliness seems to be most common in adolescents and the elderly, with 80% of teens younger than 18 and 40% of adults over 65 reporting loneliness at least sometimes (3). Notably, loneliness may affect individuals in an age-dependent manner: a large combined longitudinal study investigating the effect of social relationships on health across the lifespan found, for example, that social isolation significantly increased the risk of abdominal obesity in adolescents, but hypertension in older adults (26). Despite these differences, the physiological impacts of loneliness emerge in adolescence and persist through life (26).

### Psychosocial and behavioral changes in loneliness

Individuals experiencing prolonged loneliness exhibit a range of psychological and behavioral changes. Feelings of unsafety, which stem from loneliness, result in a chronic hypervigilant state, leading to increased anxiety, altered stress responsiveness, and social withdrawal (3, 21). Functional magnetic resonance imaging (fMRI) studies of the visual cortex and ventral striatal area revealed that lonely individuals are more likely to pay attention to and remember negative interactions and derive less reward from social stimuli (3). Importantly, these negative cognitive biases in lonely individuals can thus perpetuate social isolation in a vicious cycle in which loneliness begets loneliness, irrespective of the presence of metabolic or psychiatric conditions. Lonely individuals get less salubrious sleep, have diminished executive function compared to non-lonely individuals, and are more likely to engage in impulsive and unhealthy behaviors (3, 18, 21, 27). Further, cross-sectional and longitudinal research shows that lonely individuals show poorer self-regulation and have fewer odds of engaging in regular exercise than non-lonely individuals (28).

In addition to exercise and poor sleep, loneliness is associated with substance use disorder (SUD). Though no substantial differences in prevalence or severity of loneliness are seen across users of different substances, higher severity and duration of substance dependence consistently predicts higher levels of loneliness (29). Whether cognitive patterns that predispose to loneliness overlap with those predisposing to SUD remains unclear, and longitudinal studies have yielded mixed results regarding the causal direction or dynamics of association between them (29). Rather, there appears to be a mutually reinforcing relationship between loneliness and SUD. On one hand, social stress, social isolation, and feelings of loneliness have consistently been identified as risk factors for the development and progression of SUD (29, 30). On the other hand, stigma associated with SUD, disruption of support networks in acute stages of the disease, and foregoing of old detrimental relationships in people recovering from SUD have all been found to further feelings of loneliness (29, 31). Regardless, loneliness remains intricately tied to substance use disorder.

Chronic loneliness may thus predispose individuals to metabolic and mental health disorders through increased stress sensitivity, reduced sleep quality, and less health-promoting behaviors, including substance misuse.

### Neuroendocrine changes in loneliness

#### Hypothalamic-pituitary-adrenal axis in loneliness

The brain is the central organ for appraising and responding to psychosocial stressors like loneliness through the activation of neuroendocrine stress pathways such as the HPA and sympathetic-adrenal-medullary (SAM) axes (32), which exert broad effects on the body through glucocorticoid (cortisol in humans) release by the adrenal cortex and catecholamine release by the adrenal medulla, respectively. Circulating glucocorticoids (GC) constrain HPA axis activity through feedback inhibition and act on virtually every cell type in the body by binding to intracellular glucocorticoid receptors (GRs), which then migrate to the nucleus to regulate the transcription of hundreds of genes involved in glucose metabolism and inflammatory signaling (21). Although some studies suggest that loneliness also leads to SAM axis overactivation, findings remain less numerous and inconsistent relative to the literature on the HPA axis, which might be in part attributable to the role of SAM axis as modulator of the short-term response to stress (21).

Studies of loneliness and chronic social isolation in humans and animal models have consistently found an overactivation of the HPA axis, supported by findings of higher cortisol awakening responses (CAR), greater total GC output (area under the curve; AUC), and flattened diurnal cortisol rhythms in lonely individuals (33–39). Persistently high cortisol levels are associated with far-reaching physiological consequences, including hyperglycemia, increased vascular resistance, redistribution of body fat to the viscera, and accelerated biological aging (40). These changes can directly lead to insulin resistance and hypertension and thus represent a mechanism by which loneliness may drive the development of metabolic syndrome.

Several studies also suggest that prolonged HPA hyperactivity in loneliness is associated with greater GC resistance and thus, a corresponding disinhibition of proinflammatory gene expression (21, 34, 41, 42). DNA microarray analyses of circulating leukocytes from lonely individuals revealed an overexpression of NF-κB/Rel-driven genes involved in immune activation and cell proliferation, and an under-expression of anti-inflammatory glucocorticoid response elements, relative to socially connected controls (43). Indeed, loneliness has been associated with the up-regulation of pro-inflammatory cytokines like IL-6, IL-8, and TNF-a, as well as inflammatory transcriptional profiles in monocytes and microglia (42, 44). It is well-established that increased inflammation may contribute to the development of cardiometabolic diseases like T2DM and atherosclerosis, as well as mental health disorders like anxiety and depression (41). Perhaps as a consequence of these immunometabolic changes, loneliness has been linked to impaired humoral and cellular immunity as supported by weaker antibody response to flu vaccination, increased antiviral antibodies, and diminshed natural killer (NK) cell activity among individuals experiencing loneliness (3, 21, 42, 45–47). Thus, HPA axis overactivity and consequent glucocorticoid resistance in individuals experiencing loneliness can result in chronic inflammation and potentially lead to related sequalae, like conditions of MetS.

#### Oxytocin as a mediator between social connection and the HPA axis

While loneliness and social isolation may activate the HPA axis as a stressor, social connection may dampen this activation and impart anti-stress effects through the release of oxytocin. Oxytocin is released within the hypothalamus during positive social interactions and acts as both a neurotransmitter and hormone with far-reaching effects on the body (48). Importantly, oxytocin has a well-established role in suppressing HPA activity by inhibiting the release of corticotropin-releasing hormone from hypothalamic neurons. Thus, the absence of social interactions may increase HPA activation through a decrease in oxytocin-mediated neurotransmission. In animal studies, the direct administration of oxytocin protected against the behavioral and physiological effects of isolation (49). Beyond its action on the HPA axis, oxytocin also has well-established direct cardioprotective and neuroprotective effects as an anti-inflammatory and antioxidant hormone (50–52).

### Mitochondrial dysfunction as a target and effector of stress signaling in loneliness

Mitochondria are both sources and targets of stress signals and thus play a critical role in both regulating and carrying out the stress response (53). They are sensitive to chemical stress mediators, including glucocorticoids and oxytocin, and in response, modulate physiological adaptations to stress by releasing glucocorticoids and mitokines (54). These chemical adaptation signals of mitochondrial origin epigenetically regulate bioenergetic processes that make the stress response possible (55). Prolonged subjection to stressful stimuli and their corresponding metabolic oversupply leads to the accumulation of mitochondrial damage and disruption of structural and functional integrity, eventually reaching a threshold in which the energetic demands of the stress response can no longer be met efficiently (54, 56). For instance, sustained exposure to high GC levels diminishes mitochondrial calcium-buffering capacity, induces pathological production of reactive oxygen species, reduces mitochondrial membrane potential, alters mitochondrial morphology, and dysregulates mitochondrial fusion and fission (57, 58). This overall dysregulation of mitochondrial function from cumulative stress-related damage is termed mitochondrial allostatic load (MAL) (59). Social disconnection is associated with increased MAL through HPA overactivation in animal models (60).

The brain, consuming 20% of body energy despite accounting for 2% of total body mass (61), is highly susceptible to dampened energy production. Robust evidence from pharmacologic, genetic, molecular, and neuroimaging studies in both animal models and human’s links mitochondrial dysfunction with psychiatric disorders [for a review, see (62)]. Elevated levels of circulating cell-free mitochondrial DNA (ccf-mtDNA), the most prominent marker of mitochondrial damage, have been associated with mental health conditions, including MDD (63). Compared to controls, individuals who recently attempted suicide were found to have higher ccf-mtDNA, with levels that correlated with HPA-axis hyperactivity (64). Translation abnormalities and alterations in mtDNA sequence and copy number have been found in individuals with MDD, bipolar disorder, substance use disorder, and schizophrenia (65).

Given their central role in cellular metabolism, the implication of mitochondrial dysfunction in the etiology of metabolic diseases is unsurprising but notable, with persistent hyperglycemia serving as an illustrative example. Chronic states of elevated blood glucose–whether caused by chronic stress, inflammation, or overnutrition–are associated with the accumulation of mtDNA damage and fragmented mitochondria (66, 67). Correspondingly, mtDNA mutational burden is associated with age-related chronic disease; mtDNA mutations typically increase with age, especially in patients with diabetes (68). Mitochondrial dysfunction, such as impaired energy production and altered dynamics, has been extensively described in diabetic cardiomyopathies (69), one of the main causes of death for patients with MetS. Similar associations between mtDNA damage and other conditions of MetS have been described extensively elsewhere (70–72).

**FIGURE 1:**
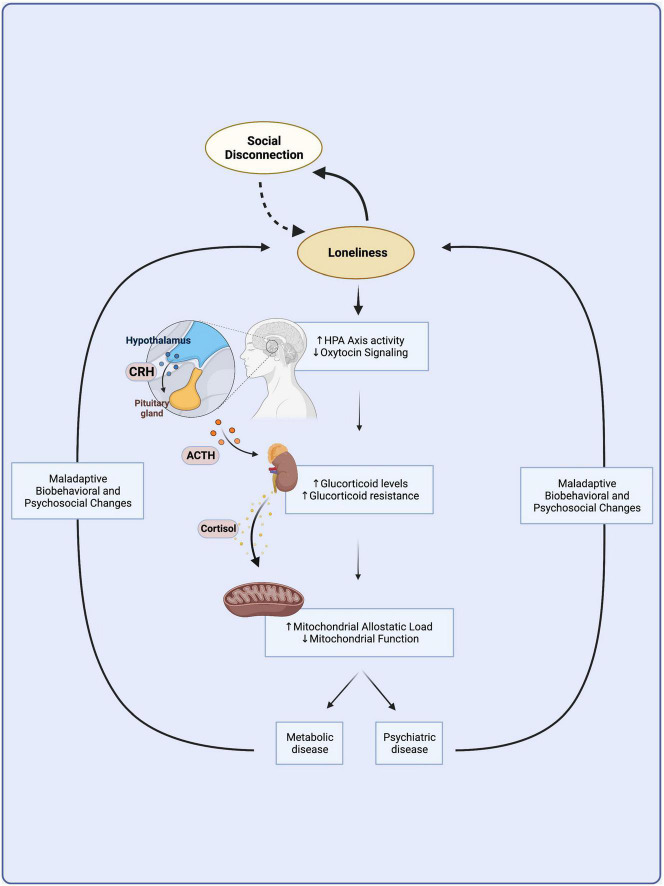
A vicious cycle links loneliness with metabolic and psychiatric disease. Loneliness arises from social disconnection as the discrepancy between desired and actual levels of social connection. Loneliness might lead to further social isolation through maladaptive biobehavioral changes and increased stress sensitivity in a vicious cycle independent of disease. Chronic loneliness is a prolonged psychosocial stressor that becomes embedded in the body through the overactivation of the hypothalamic-pituitary-adrenal axis, which leads to chronically elevated levels of circulating glucocorticoids. Chronic HPA axis hyperactivity leads to glucocorticoid resistance and mitochondrial dysfunction that culminate in immunometabolic changes common to the etiologies of many metabolic and mental illnesses. Biobehavioral and psychosocial changes observed in metabolic and mental illness might contribute to a vicious cycle furthering loneliness and its adverse downstream neuroendocrine and immunometabolic consequences. HPA, hypothalamic-pituitary-adrenal; CRH, corticotrophin releasing hormone; ACTH, adrenocorticotropic hormone. Created with BioRender.com.

To summarize, the long-term experience of loneliness becomes embedded in the body through chronic stress at the biobehavioral, neuroendocrine, and mitochondrial levels, impeding metabolic function and contributing to the onset of physical and mental chronic diseases (Figure 1).

## Loneliness leads to a vicious cycle of illness

Loneliness is not only a cause but also a consequence of mental health and metabolic disorders. The causal pathways linking poor metabolic and mental health to social disconnection and loneliness are likely better understood at the biobehavioral and psychosocial levels.

A brief examination of depression illustrates how experiencing certain mental health disorders may lead to further social isolation and worsening feelings of loneliness. For example, when establishing novel relationships in the setting of an emerging social group, depressive symptoms at baseline predict fewer social interactions, more time spent with similarly-depressed individuals, and prioritizing novel dyadic interactions over broader group interactions (73). Once friendships are established, the ability to maintain positive relationships and terminate negative ones might be impaired (74). Difficulties in generating and maintaining social connections in depression might be explained by increases in rejection sensitivity, maladaptive social cognition, decreases in social self-efficacy, and disruptions in social skills, among other behaviors and predispositions (75–78). The stigma surrounding mental health might further exacerbate ostracism among individuals experiencing a mental health condition.

Beyond their association with psychiatric disease, considering certain conditions of MetS and potential mediators of their association with social isolation can begin to shed light on plausible mechanisms at a sociobehavioral level. For example, when compared to other individuals with obesity, those experiencing higher internalized weight bias and more frequent experiences of weight-related discrimination feel more lonely (79). This evidence suggests that obesity might lead to social isolation and subsequently loneliness through socio-normative pressures and the internalization of weight-related stigma (16). Other conditions of MetS, like CVD, might lead to social disconnection by reducing health fitness and limiting the individual’s ability to socialize through sports and physically-demanding activities, a possibility that should be considered given the remarkable effects of group exercise on social connectivity and prosocial behavior (80). More generally, if the morbidity from conditions of the MetS is severe enough to lead to substantial disability and need for involved care, the strain placed on caregivers might lead to disruption of key social relationships central to the patient’s wellbeing.

Thus, biobehavioral and psychosocial maladaptive changes associated with mental health conditions and poor metabolic health might expose individuals to acute social isolation, predispose individuals to fail to adapt to this acute stressor, and facilitate the progression and maintenance of the chronic stress of loneliness.

## Interventions for treating and preventing loneliness

Akin to a viral pandemic, loneliness spreads through social networks *via* a contagious process (81, 82). Critically, loneliness appears in clusters of people closely interacting with one another and is more severe at the periphery of social networks (81). These observations point to the multiplicative public health impact interventions might have if they lead to the strengthening and conservation of social connections, even at the individual and small community level. Thus, addressing loneliness resulting from widespread social disconnection emerges as an actionable, cost-effective, and influential target for intervention.

Keeping the chronic stress paradigm laid out above in mind, two general approaches to prevent the progression from social disconnection to loneliness and, furthermore, address the deleterious downstream effects of loneliness are (1) building psychological and bioenergetic capacity to adequately respond and adapt to stress and (2) removing or minimizing the stressor. While the former can be achieved most effectively through interventions at the individual level, the latter calls for interventions at the community and societal level. By building stress resilience at the individual level, increasing social connectedness at the community level, and advancing policies that support a public health infrastructure that increases connectedness at a societal level, the interventions recommended below might help break the vicious cycle linking social disconnection to the development and progression of metabolic and mental health disorders (Table 1).

**TABLE 1:** Interventions for treating and preventing loneliness.

Building stress resilience	Increasing social connectedness
**Mental resilience**	**Community level**
Practicing self-reflection	Seeking collective effervescence
Improving interoception	Religious gatherings
Improving stress management	Concerts and music festivals
Diaphragmatic breathing	Sports events
Meditation	Political demonstrations
Loving-kindness	Seeking shared experiences
Compassion	Peer support groups
Fomenting adaptive appraisals	Communal meals
Practicing self-compassion	Community gardening
Gratitude practices	House renewal
Cognitive strategies–including cognitive behavioral therapy	Visiting nature
Avoid upward contrast	Digital interventions
Considering role of FoMO	Other group activities
Increasing oxytocin signaling	**Policy level**
Skin-to-skin stimulation	Medicine
Gustatory stimulation	Loneliness screening guidelines
Positive human interactions	Support infrastructure to address loneliness
Interaction with pets	Social prescribing
**Metabolic resilience**	Increasing social connectivity
Increasing movement	Physical connectivity
Optimizing diet and nutrition	Digital connectivity
Ketogenic diet	Encouraging corporate wellness efforts
Intermittent fasting, caloric restriction	Spearheading educational interventions
Minimizing exposure to POPs	Address maladaptive social cognition

Interventions that (1) increase bioenergetic and psychosocial capacity to respond to stress and (2) remove the stressor of social disconnection will be protective against loneliness. By focusing on building metabolic and mental stress resilience at an individual level, increasing social connectedness at the community level, and prioritizing public policies that support society’s key institutions in connecting the people they serve, we can begin to address the growing epidemic of loneliness and break the cycle linking it to poor metabolic and mental health.

### Individual level: Building stress resilience by increasing psychological and bioenergetic capacity to respond to social disconnection

#### Mental resilience

Whether social disconnection turns into loneliness and the extent to which this perceived social isolation persists over time is ultimately determined by the psychological appraisal of our situation in relation to the rest of the world. Among other things, this process relies on self-efficacy, the subjective interpretation of environmental cues, interoceptive interpretations of internal sensations, the processing of feelings and emotions as they relate to social interactions, and belief systems and expectations surrounding the number and quality of social connections we ought to have (83–86). Hence, independent of objective changes to one’s level of social connection, building mental resilience will protect against loneliness by facilitating adaptive appraisals of our place in relation to the rest of the world.

Practices that encourage self-reflection, like journaling, can help in identifying stress arising from loneliness by improving interoception and increasing self-awareness and emotional regulation (84). Journaling has been associated with decreased mental distress among general medical patients with anxiety symptoms in a preliminary randomized controlled trial (87). Once the stress state is identified, deploying relaxation and stress management techniques known to dampen the stress response, like diaphragmatic breathing or loving-kindness and compassion meditation, might improve emotional regulation and help prevent the progression of maladaptive physiological processes tied to loneliness (88, 89). Indeed, unlike control subjects, individuals practicing diaphragmatic breathing show reductions in cortisol levels following a single session (89). In a classic study, Fredrickson et al. found that practicing loving-kindness meditation is associated with increased social support in the long term, hinting at its potential value in addressing loneliness specifically (88).

Daily gratitude practices and writing exercises that encourage self-compassion predispose individuals to more adaptive appraisals of future social interactions (90). For example, self-compassion is associated with greater equanimity when resolving future relationship conflicts (91). Cognitive strategies tied to reductions in loneliness include avoiding social comparisons leading to upward contrast (85) and reflecting on the role of fear of missing out as a contributor to loneliness (92). Although several systematic reviews have highlighted the need for more rigorous assessments of the efficacy of interventions against loneliness, a meta-analysis of 50 studies including young and older adults concluded that interventions combatting maladaptive social cognition, including multiple forms of cognitive behavioral therapy, are more effective than strategies focusing on increasing social skills and enhancing social support at reducing loneliness (93).

Experiences that increase oxytocin signaling increase stress resilience by contributing to the development and maintenance of the body’s neuroendocrine stress buffering capacity. Examples of interventions found to increase oxytocin signaling include skin-to-skin stimulation like massages, gustatory stimulation, positive human interactions like cohabitation and safe, consensual sexual interactions, and interaction with pets (48). Notably, a systematic review and meta-analysis of 44 studies found that animal therapy is more effective than psychotherapy and occupational therapy, among other types of interventions, in reducing loneliness among older adults (94).

#### Metabolic resilience

The response to the stress arising from social disconnection, whether adaptive or maladaptive, is ultimately instantiated by the mitochondria. Mitochondrial dynamics involved in the stress response are energy-demanding, particularly when stress-buffering strategies at the psychosocial level are insufficient to prevent the progression from an adaptive response to stress arising from social disconnection to the chronic stress response tied to loneliness. Thus, interventions that build metabolic fitness, or mitochondrial bioenergetic capacity, will build stress resilience.

Movement, in the form of endurance exercises (95), resistance training (96), or yoga (97) induce protective changes in biogenesis, fusion rates, volume, structure, and function of mitochondria, increasing the body’s overall bioenergetic capabilities (98). Notably, these changes, even when occurring most evidently within skeletal muscle, are also thought to underlie exercise-induced neuroprotection (99). At an epidemiological level, higher levels of physical exercise positively predict prosocial behavior among children and adolescents (100, 101).

Dietary changes that facilitate the maintenance of healthy blood glucose levels and help avoid chronic overnutrition will optimize mitochondrial function and build stress resilience. In particular, the ketogenic diet (KD), a high-fat, low-carbohydrate diet mimicking the metabolic state of starvation, improves several markers of mitochondrial redox status by inducing the production of antioxidants and detoxification enzymes (102, 103). Along those lines, caloric restriction and intermittent fasting (IF) induce protective changes in mitochondrial dynamics, reduce mitochondria-related oxidative stress, and improve the energetic output of mitochondrial respiration (104, 105). Reducing exposure to persistent organic pollutants (POPs), for example, by preferring organic food and filtering water and air, may further prevent mitochondrial damage (106).

### Community level: Minimizing the stress of loneliness by increasing social connectedness

Beyond building stress resilience, seeking experiences that decrease loneliness by either increasing the number and quality of social connections, or a sense of connectedness to the world around us, will contribute to better mental and metabolic health. Here, we list a number of ways to increase connectedness at the community level.

Higher frequency of experiences of collective effervescence, or the sensation of shared sacredness and connection arising in group gatherings, confers significant protection against loneliness (107, 108). Although originally conceived as arising from religious gatherings, it is now understood that collective effervescence is also commonly brought about by more ubiquitous group experiences, including concerts, sports events, and political demonstrations (107, 109). Concerts and music festivals can further increase feelings of connection to other attendees through joint action and interpersonal coordination synchronized to the music rhythm (110).

Not surprisingly, shared experiences at the community level, even in the absence of collective effervescence, lead to reductions in loneliness (111, 112). Support groups, particularly those centered around common experiences of illness and peer-to-peer support, can increase feelings of connectedness while offering practical advice (113–115). In fact, a systematic review by Cattan et al. concluded that group interventions, particularly those targeting a specific group with a shared identity or experience, might be more effective at reducing loneliness than one-on-one strategies (116). Examples of other group community efforts that likely reduce loneliness include participating in communal meals and community gardening or house renewal projects, visiting nature sites with friends, and taking art or cooking classes (112, 117).

Digital interventions have gained popularity after the onset of the COVID-19 pandemic. Although still limited in their reach by disparities in access to technological resources such as high-bandwidth internet associated with lower socioeconomic status, certain digital interventions have proved to be effective at reducing loneliness across a wide range of demographic groups during the COVID-19 pandemic (118–120). For instance, in a series of four studies including over one thousand participants involving younger and older adults in Australia and the United Kingdom, GROUPS 2 CONNECT, a web-based intervention deploying a series of interactive screens inviting participants to engage in priority- and goal-setting around social relationships, was found to lead to improvements in self-reported quality of social connections and ability to stay connected over time (121). As digital interventions against loneliness will likely become a mainstay in the post-pandemic era, priority should be given to those that (1) take provisions to minimize accessibility concerns, (2) are developed based on a robust theoretical framework (122), (3) facilitate more frequent direct and meaningful (as opposed to fleeting) interactions among individuals (123), and (4) include a technology education component when targeting older adults (124).

### Societal level: Minimizing the stress of loneliness by advancing community-building public policy

Public policies directly aiming to address loneliness can support society’s key institutions—including health care systems, workplaces, religious and secular community organizations, schools, and colleges—in being more intentional and systematic about connecting the society members they serve.

In health care, screening for loneliness with objective measures at primary care appointments and developing an infrastructure for connecting individuals to programs, resources, and institutions focusing on addressing social disconnection should be considered. Currently, despite the negative health consequences of social isolation–which is comparable to risk factors that are screened routinely, like heavy drinking or smoking (125)–guidelines recommending screening for loneliness in the primary care setting are notoriously missing. Though standardized measures of social isolation and loneliness have been developed and are often used in research (i.e., Lubben Social Network Scale, Duke Social Support Index, UCLA Loneliness Version Scales), they are not currently used in the clinical care setting, in part due to a paucity of research on their efficacy as a preventive tool in the clinical setting. Moreover, whether any of these measures is robust enough to capture loneliness as a multidimensional construct remains an area of concern (29).

In addition to promoting the advancement of evidence-based screening guidelines, public health efforts should be devoted to developing and supporting interventions that bolster social connectivity among at-risk groups. Though further research is needed, social prescribing, or the use of non-clinical referral options by clinicians, is emerging as an effective paradigm for connecting individuals to programs, resources, and institutions focused on addressing loneliness (125, 126).

Increasing access to safe and adequately lit parks, bike lanes, public transportation, recreational activities, and high-speed internet access are all ways communities can enable physical and digital proximity that can facilitate social connection for its residents (127).

A variety of corporate wellness programs improve employees’ physical and psychological health by fostering social connection in the workplace (128, 129). Policies that encourage the development and maintenance of such programs should be considered a public health priority.

Public health interventions with an educational focus might have a role in counteracting cognitive biases that further compromise social connection. In fact, educational interventions focused on addressing maladaptive social cognition are among the most effective types of interventions against loneliness (93). Such public education campaigns could be developed and implemented in partnership with K-12 schools, colleges, senior centers, workplaces, and government agencies.

## Conclusion

Loneliness is a growing public health problem at the heart of the epidemics of mental health-related conditions and metabolic health-related conditions. Although not often considered a serious risk factor for chronic disease, loneliness is a major predictor of all-cause mortality and is associated with the development of both mental and cardiometabolic disease. The chronic stress brought about by loneliness may cause HPA axis hyperactivity and adverse downstream immunometabolic consequences fundamentally arising from mitochondrial dysfunction that may ultimately lead to further social isolation. Given its role in the etiology of the most prevalent chronic diseases of our time, alleviating loneliness is a vitally important and cost-effective public health strategy, and loneliness checks should be incorporated into routine patient care. Future work should identify scalable, evidence-based interventions to reduce loneliness and its deleterious health consequences.

## Data availability statement

The original contributions presented in this study are included in the article/supplementary material, further inquiries can be directed to the corresponding authors.

## Author contributions

IC and MA contributed equally to the research and production of this manuscript from the conception and design to the research and writing. MM contributed to the conception of the original idea and provided the expert guidance on the theoretical framework for this manuscript. All authors contributed to the manuscript revision, read, and approved the submitted version.
